# A novel molecular toolkit for rapid detection of the pathogen and primary vector of thousand cankers disease

**DOI:** 10.1371/journal.pone.0185087

**Published:** 2018-01-05

**Authors:** Emel Oren, William Klingeman, Romina Gazis, John Moulton, Paris Lambdin, Mark Coggeshall, Jiri Hulcr, Steven J. Seybold, Denita Hadziabdic

**Affiliations:** 1 Department of Entomology and Plant Pathology, University of Tennessee, Knoxville, TN, United States of America; 2 Department of Plant Sciences, University of Tennessee, Knoxville, TN, United States of America; 3 USDA Forest Service, West Lafayette, IN, United States of America; 4 School of Forest Resources and Conservation, University of Florida, Gainesville, FL, United States of America; 5 USDA Forest Service, Davis, CA, United States of America; Natural Resources Canada, CANADA

## Abstract

Thousand Cankers Disease (TCD) of *Juglans* and *Pterocarya* (Juglandaceae) involves a fungal pathogen, *Geosmithia morbida*, and a primary insect vector, *Pityophthorus juglandis*. TCD was described originally from dying *Juglans nigra* trees in the western United States (USA), but it was reported subsequently from the eastern USA and northern Italy. The disease is often difficult to diagnose due to the absence of symptoms or signs on the bark surface of the host. Furthermore, disease symptoms can be confused with those caused by other biotic and abiotic agents. Thus, there is a critical need for a method for rapid detection of the pathogen and vector of TCD. Using species-specific microsatellite DNA markers, we developed a molecular protocol for the detection of *G*. *morbida* and *P*. *juglandis*. To demonstrate the utility of the method for delineating TCD quarantine zones, we tested whether geographical occurrence of symptoms and signs of TCD was correlated with molecular evidence for the presence of the cryptic TCD organisms. A total of 1600 drill cores were taken from branch sections collected from three regions (*n* = 40 trees for each location): California-*J*. *hindsii* (heavy disease incidence); Tennessee-*J*. *nigra* (mild disease incidence); and outside the known TCD zone (Missouri-*J*. *nigra*, no record of the disease). California samples had the highest incidence of the TCD organisms (85%, 34/40). Tennessee had intermediate incidence (42.5%, 17/40), whereas neither organism was detected in samples from Missouri. The low cost molecular protocol developed here has a high degree of sensitivity and specificity, and it significantly reduces sample-processing time, making the protocol a powerful tool for rapid detection of TCD.

## Introduction

In the past two decades, Thousand Cankers Disease (TCD) has caused widespread mortality of *Juglans nigra* L. (eastern black walnut) in the United States (USA) [1–4]. The disease complex effects two tree genera within the Juglandaceae, *Juglans* (walnut/butternut) [4–6] and *Pterocarya* (wingnut) [7]. TCD involves a fungal pathogen of the phloem, *Geosmithia morbida* M. Kolařík, E. Freeland, C. Utley, & N. Tisserat (Ascomycota: Hypocreales: Bionectriaceae) [2, 8] and a primary insect vector, the walnut twig beetle, *Pityophthorus juglandis* Blackman (Coleoptera: Curculionidae: Scolytinae)[4, 9, 10]. Symptoms and signs of TCD include yellowing and wilting of the leaves (flagging); crown thinning due to branch dieback; epicormic branching from the main stem; formation of numerous entrance/emergence holes through the bark and egg galleries in the phloem by *P*. *juglandis*; appearance of sunken stain spots on the bark surface; and the development of small, dark brown cankers beneath the outer bark [2, 5]. *Geosmithia morbida* grows and sporulates inside *P*. *juglandis* galleries, and although the pathogen can be dispersed by wind and water, the most efficient disease vectors are adults of *P*. *juglandis* [2, 11]. By itself, *G*. *morbida* is considered a weak and non-systemic pathogen. However, high levels of inoculation into host trees by *P*. *juglandis* can result in tree mortality [2, 4, 12]. Walnut tree die-off is usually observed three to four years after the initial disease symptoms and signs are observed [2, 12].

Though all *Juglans* species tested so far have shown some degree of susceptibility towards *G*. *morbida*, infections appear to be more severe in *J*. *nigra* [8]. This is particularly troubling since black walnut wood is highly prized for its woodworking and high-quality timber characteristics [13]. In 2009, the value of all standing *J*. *nigra* in the USA was estimated to exceed $539 billion [14]. Furthermore, the nuts of *J*. *nigra* are an important food resource for forest animals [1], and those of *J*. *regia* L. (English walnut) are a valuable commercial agricultural product [14]. The value of walnut and walnut-derived products has generated a very active wood and nursery trade among the states in the USA and among countries [12], increasing the likelihood of the spread of TCD.

In 2010, TCD was reported in Knoxville (Tennessee), which represented the first recorded occurrence in the native range of *J*. *nigra* [1] and presented an emerging threat to the highly valuable native timber stands of this species in the eastern USA [9]. In Tennessee alone, the value of *J*. *nigra* is estimated at $1.37 billion in urban areas and $1.47 billion in forest areas, underscoring its economic importance in the region [15–17]. Currently, TCD has been reported in seven eastern U.S. states: Indiana, Maryland, North Carolina, Ohio, Pennsylvania, Tennessee, and Virginia [1, 12, 18, 19]. TCD has also been reported in northern Italy [20], which is worrisome since this region is in the native range of *J*. *regia* [21]

To prevent the spread of TCD within the USA, several county and state quarantine measures are in place [22]. However, concerns remain about the efficacy of these measures due to difficulties in early detection, accurate diagnosis, and the capacity to find both organisms in the field [15, 23]. To confirm the presence of the disease in newly affected areas, some plant pest regulators require detection of both organisms in the field. Pheromone-baited traps have been effective in detecting newly established populations of *P*. *juglandis*, but the traps have to be situated relatively close to infested host material [24]. In newly colonized areas, low population densities of *P*. *juglandis* and/or *G*. *morbida* may not lead immediately to outwardly obvious symptoms and signs of TCD in trees. Specifically, TCD is often challenging to identify because the entrance/emergence holes of *P*. *juglandis* are very small (0.2–0.4 mm diameter); the bark surface may not always exhibit any symptoms or signs of *G*. *morbida* infection; and crown symptoms can be confused with drought damage [12, 23]. Most TCD symptoms and signs first become apparent long after the tree has been colonized by the beetle and the fungus. Even when symptoms and signs are obvious, morphological confirmation of *G*. *morbida* in culture can be challenging, time-consuming, and often unreliable. Currently, a TCD-specific nutrient medium is not available, which is one of the biggest challenges in recovering *G*. *morbida* from infected tissues [23]. Furthermore, other, faster growing fungi in the necrotic areas, galleries, or potentially living as endophytes [25] can outcompete *G*. *morbida* and prevent confirmation of the pathogen through traditional methods. Lastly, culture-based methods involve multiple isolations from infected material (inner bark and wood), multiple re-isolations to obtain suspected *G*. *morbida* axenic cultures (2–6 weeks), and the microscopic identification of the pathogen. Diagnostic validation of the fungus and beetle requires trained personnel with expertise in fungal or insect taxonomy as well as fungal culturing.

Molecular techniques can provide valuable tools in plant disease diagnosis and can be used for the simultaneous detection and identification of plant pathogens [26]. PCR-based methods have become the primary means for the early detection of pathogens in agriculture and forestry [27], as well as in human and animal health [28]. Molecular methods are sensitive enough to detect minute amounts of DNA from the target organism even when the substrate is heterogeneous, thereby decreasing the time needed to isolate and confirm the presence of the pathogen of interest [26, 27, 29, 30]. With the goal of reducing the time invested in the detection of both organisms involved in TCD, this study developed a protocol that utilizes species-specific microsatellite loci as molecular tools for their rapid detection. We have tested the utility of the method by using a continental-scale sample of tree host tissues from locations affected differentially by TCD, and tested whether the presence or absence of disease symptoms correlate with the presence/absence of the cryptic causal agents.

## Materials and methods

### Collection of walnut branches

A total of 120 *Juglans* spp. branch sections (~ 5–8 cm diameter and 35–45 cm long) were collected from live trees from three separate locations, either within a heavily impacted area [California (Yolo County, Putah Creek Ecological Reserve—*J*. *hindsii*)], mildly impacted area [Tennessee (across the following counties Blount, Knox, Loudon, Marion, Morgan, Rhea, and Sequatchie—*J*. *nigra*)], or outside of the TCD-confirmed areas [Missouri (Howard County, University of Missouri Horticulture and Agroforestry Research Center—*J*. *nigra*)]. Forty branch sections were collected from each location and were stored at 4°C until processed. Two different sampling approaches were used to obtain wood shavings from the branches: (1) lesion-directed (n = 1200) for tree tissue with symptoms or signs suggesting either target organism, and (2) feature-directed (n = 400) for asymptomatic tree tissue where wounds or other potential entry points were sampled. A total of 1600 drill cores were taken, and shavings from these cores were used for DNA extraction.

### Sampling process

#### Lesion-directed sampling

For lesion-directed sampling, bark was removed from each branch section with a sterilized scalpel, exposing cankers and discolored phloem. Preliminary observations of *G*. *morbida*-induced lesions have revealed a broad diversity in phloem discoloration, ranging from tan and dark brown hues to nearly black tissues. Ten cankers per branch section were identified and marked for subsequent drilling. Drill bits (7/64 inch–0.28 cm diameter) were heat sterilized between drillings with a bead sterilizer (Steri 350, Fisher Scientific, Pittsburgh, PA, USA) to prevent cross-contamination. The sterilized drill bit was placed through a microfunnel (bottomless microcentrifuge tube) and the canker was punctured, potentially contacting *G*. *morbida* hyphae and *P*. *juglandis* body parts. Wood shavings were collected inside the microfunnel and the process was repeated until ~ 150 mg of material was obtained. All drill shavings from a sample were transferred to a sterile 1.5 ml safe-lock microcentrifuge tube and stored at -20°C until processed. A total of 10 lesion-directed samples were taken from each branch section for a total of 1200 drilled samples. Branch sections from Missouri (area with no known TCD) were used as a negative control, and we targeted lesions in the phloem, discolored areas of the outer bark tissues, and bark wounds.

#### Feature-directed sampling

To test the specificity of the method and to simulate field conditions, an additional set of five feature-directed samples were taken from each branch section. This sampling approach was performed to emulate the collection of samples from potentially asymptomatic trees under field conditions, where no stereomicroscope or other laboratory tools (i.e., scalpels) are available and consequently cankers cannot guide the drilling process. Therefore, samples were taken from the vicinity of natural (lenticels) or insect-generated openings; secondary branch unions; bark furrows; and various other bark crevices and wound sites without bark removal. Drilling and sample processing methodology were the same as in lesion-directed sampling. Feature-directed samples were taken from branch sections collected in TCD-confirmed locations from both symptomatic and asymptomatic trees in California and Tennessee (*n* = 400).

#### DNA extraction and PCR

DNA from the wood shavings was extracted by using either a QIAamp Fast DNA Stool Mini Kit or a DNA Stool Mini Kit (QIAGEN, Germantown, MD, USA), following the manufacturer’s suggested protocol. PCR amplifications were performed by using a *G*. *morbida*-specific microsatellite locus (GS 004) [31] and a *P*. *juglandis*-specific microsatellite locus (WTB 192) [32] that yield peaks at different positions based on size. These markers were selected on the basis of preliminary data assessments for these marker’s capabilities through which, GS 004 and WTB 192 yielded the highest rate of amplification. PCR reactions contained 1 μL DNA (undiluted, concentration not determined), 4 μl Go-Taq G2 Hot Start Colorless Master Mix (Promega, Madison, WI, USA), 1 μL of 10 μM of each primer, 0.5 μl DMSO and 3.5 μL of sterile water for a total volume of 11 μL. Both positive and negative controls were included in each run. PCR amplifications were performed in a 96-well plate, with an Eppendorf Mastercycler pro Thermocycler (Eppendorf AG, Hamburg, Germany) and an initial denaturation step of 2 min at 95°C, followed by 30 cycles of 95°C for 30 s, 55°C for 45 s, 70°C for 1 min, and a final extension at 70°C for 1 min.

Following PCR amplification, PCR reactions were analyzed with a QIAxcel Capillary Electrophoresis System (QIAgen, Valencia, CA, USA) with the inclusion of a 25 base pair DNA marker. The QIAxcel system uses ScreenGel software that determines the base pair number of each amplicon in individual PCR reactions and automatically generates a file containing all data gathered from the 96-well plate. Use of this software facilitates rapid data interpretation and provides flexibility in data viewing since results are displayed as electropherograms and as gel images (Fig A in S1 File).

### Cross-transferability of Geosmithia morbida and Pityophthorus juglandis microsatellite loci

#### Cross-transferability of the GS 004 microsatellite locus

To confirm the specificity of the *G*. *morbida* microsatellite locus GS 004 and to minimize the possibility of false positive samples, several fungal isolates known to co-inhabit *P*. *juglandis* galleries were examined. Cultures used for testing were collected as part of an ongoing project that aims to characterize the broader fungal community associated with TCD-symptomatic trees [33]. Briefly, diverse fungi were isolated directly from *P*. *juglandis* galleries or from surrounding necrotic areas in the phloem and xylem. For the latter, excised wood pieces were placed directly in ½ strength Difco^TM^ Potato Dextrose Agar (PDA) (Acros Organics, Fisher Scientific, Bridgewater, NJ, USA) amended with antibiotics (1% chlorotetracycline-streptomycin sulfate solution) or first incubated in moist chambers for 3–5 days. Once axenic cultures were obtained, isolates were identified by using the Internal Transcribed Spacer (ITS) region, following established protocols [34]. The same isolates, representing taxonomically diverse Ascomycota lineages, were further tested for the amplification of GS 004 (Table A in S1 File). PCR reactions and amplicon analyses were conducted following the aforementioned protocol.

#### Cross-transferability of the WTB 192 microsatellite locus

Twenty-four bark or woodboring beetle species (Table B in S1 File), either closely related to *P*. *juglandis*, or frequently associated with walnut trees, were trapped from localities in Tennessee and Florida during 2014 and 2015. Specimens were stored in 70% ethanol until processed for DNA isolation. Prior to DNA isolation, specimens were placed for 15 min in a Petri dish containing distilled water and then transferred onto filter paper to allow for evaporation of residual ethanol. Samples were then placed in 2 ml conical screw-cap microcentrifuge tubes (Fisher Scientific) containing 2.3 mm diameter zirconia/silica beads (BioSpec Products, Bartlesville, OK, USA). Beetle tissue was lysed by using a Bead Mill 24 homogenizer (Fisher Scientific). DNA was extracted by using Thermo Scientific MagJET Genomic DNA Kit (Fisher Scientific), according to the manufacturer’s suggested protocol with a few modifications. Modifications that yielded optimal DNA recovery included: (a) an additional use of 20 μl of proteinase K/sample prior to overnight incubation at 56°C in a digital dry bath/heating block (Fisher Scientific); (b) the elution buffer, heated to 70°C, was added twice (45 μl/each elution) to the column containing DNA with 5 min incubation periods at room temperature between elutions; and (c) after each elution, samples were centrifuged for 1 min at 8000 rpm, resulting in final DNA volume of 90 μl/sample. DNA samples from species other than *P*. *juglandis* were used to confirm the specificity of the *P*. *juglandis*-specific WTB 192 microsatellite locus. PCR reactions and amplicon analyses were conducted following the aforementioned protocol.

#### Statistical analyses

Data on presence or absence were analyzed in the statistical environment R [35]. Pearson’s Chi-square test (α = 0.05) with Yates’ continuity correction was performed to test for independence of *G*. *morbida* detection with *P*. *juglandis*. A logistic mixed model analysis was used to compare detection frequencies among locations (CA, MO, TN) and sample types (lesion-directed samples or feature-directed samples), as well as the interaction between these factors using lme4 package [36], with further processing of results using lmerTest [37] and lsmeans [38], and ggplot2 [39].

## Results

**Fig 1:**
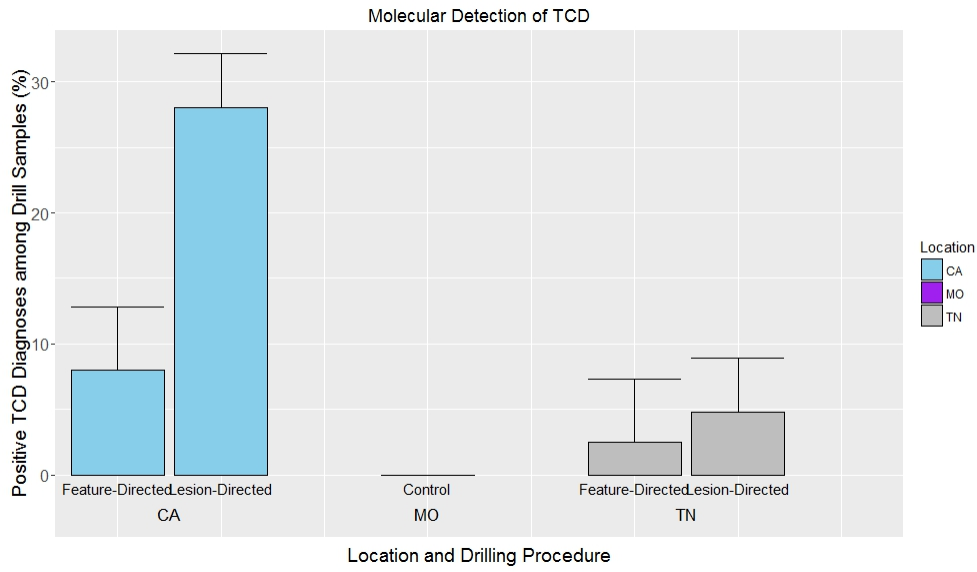
Molecular detection of thousand cankers disease (percentage of positive drill samples) from both feature-directed and lesion-directed samples from California, Missouri (control), and Tennessee.

**Fig 2:**
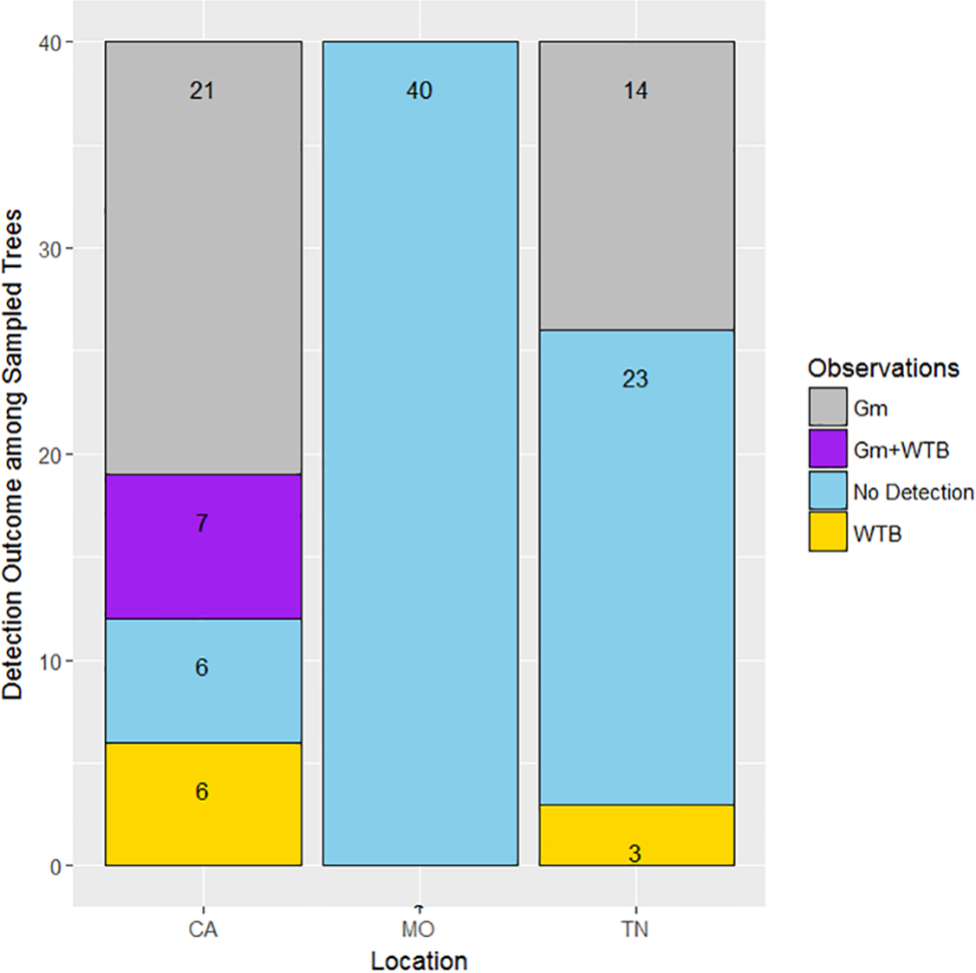
Molecular detection outcomes among 40 samples each from California (*Juglans hindsii*), Missouri (control, *Juglans nigra*) and Tennessee (*J*. *nigra*) for both drilling methods.

**Fig 3:**
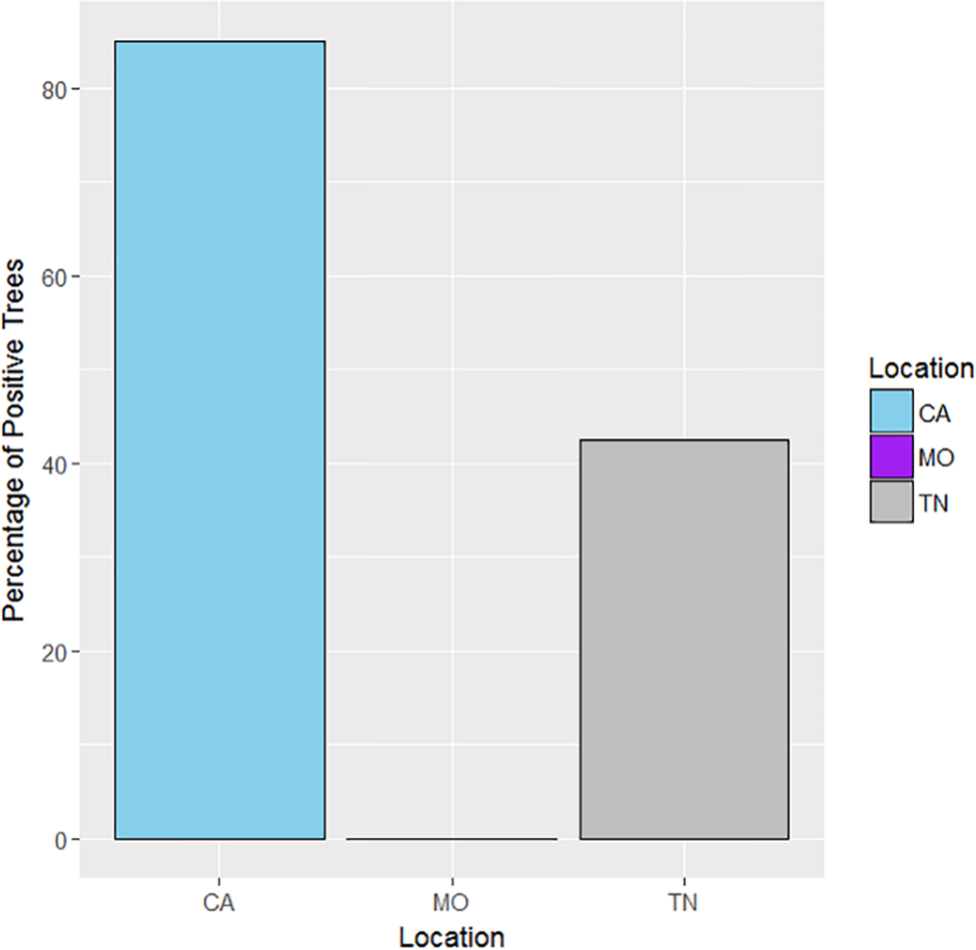
Molecular detection of thousand cankers disease organisms (*Geosmithia morbida* and/or *Pityophthorus juglandis*) among positive trees for all samples and both drilling methods.

Confirmation of the presence of the TCD organisms was based on the positive results obtained from the amplification of species-specific microsatellite loci from drilled bark samples. Efficiency of these markers in rapidly detecting TCD organisms from both lesion- and feature-directed drilling methods was inferred (Pearson’s Chi-square test with Yates’ continuity correction). The analytical cost (based on consumables) was estimated at $10.76 per sample. Detection outcomes among all sampled trees (n = 120) across the three different locations (n = 40 trees/location), confirmed that *G*. *morbida* and/or *P*. *juglandis* were detected at a higher rate in California samples than in Tennessee samples (Fig 1). The validity of the detection method was supported, as the control location (Missouri) was negative for the presence of both the pathogen and beetle in nearly all samples examined. In only a few instances with the material from Missouri was there a suggestion of positive responses in the assay for *P*. *juglandis*, but results were inconclusive (see discussion for more details). *Geosmithia morbida* was detected from 21 California samples and 14 Tennessee samples when results from both drilling methods were pooled (Fig 2). *Pityophthorus juglandis* DNA was detected exclusively from six California samples and three Tennessee samples. However, only seven California samples had both the pathogen and the beetle (Fig 2). Altogether, 85 percent (n = 34/40) of sampled *J*. *hindsii* trees in California and 42.5 percent (n = 17/40) of sampled *J*. *nigra* trees in Tennessee were confirmed to have *G*. *morbida* and/or *P*. *juglandis*, demonstrating the association of these samples with at least one member of the TCD complex (Fig 3). When data were partitioned according to the two drilling methods, 80 percent (n = 32) of *J*. *hindsii* trees and 30 percent (n = 12/40) of *J*. *nigra* trees in California and Tennessee, respectively, were confirmed for the presence of *G*. *morbida* and/or *P*. *juglandis* by using the lesion-directed method, whereas 37.5 percent (n = 15) of *J*. *hindsii* trees in California and 15 percent (n = 6) of *J*. *nigra* trees in Tennessee were confirmed for the presence of *G*. *morbida* and/or *P*. *juglandis* by using the feature-directed method.

Lesion- and feature-directed sampling yielded differences in the ability to detect TCD and varied depending on the different species-specific microsatellite loci examined [*Χ*^2^ (df = 1, n = 1600) = 14.11, *P* < 0.001] (Table 1). When the two drilling methods were analyzed separately, both lesion-directed [*X*^2^ (df = 1, n = 1200) = 14.24, *P* < 0.001] and feature-directed [*X*^2^ (df = 1, n = 400) = 11.16, *P* < 0.01] drilling procedures detected *G*. *morbida* and/or *P*. *juglandis* (Table 1). Effect of location was also assessed for both drilling methods (Table 1). When California and Tennessee locations were tested by combining data from the two drilling methods, differences in detection by the two microsatellite loci used in this study were not apparent (Table 1). However, when California samples were separated by drilling method, the results were significant [*X*^2^ (df = 1, n = 400) = 6.68 *P* < 0.001, *X*^2^ (df = 1, n = 200) = 5.99, *P* < 0.01, for lesion-directed and feature-directed approaches, respectively] (Table 1).

**Table 1:** Comparisons of molecular detection of *Geosmithia morbida* (*Gm*) and *Pityophthorus juglandis* (*Pj*) among drill cores taken from three locations, including sites (CA, TN) where the organisms are known to occur^A^.

Location	Sample Type	N^B^	*Pj*^(-)^/*Gm*^(-)^	*Pj* ^((+)^/*Gm*^(-)^	*Pj* ^((-)^/*Gm*^(+)^	*Pj* ^((+)^/*Gm*^(+)^	*Χ*^2^ (df = 1)^C^	*P-*value
CA, TN, MO*	LD&FD^D^	1600	1420	28	141	11	14.11	< 0.001
CA, TN, MO	LD	1200	1065	4	126	5	14.24	< 0.001
CA, TN	FD	400	355	24	15	6	11.16	< 0.001
CA	LD&FD	600	449	23	117	11	1.96	*NS*^E^
TN	LD&FD	600	571	5	24	0	0	*NS*
CA	LD	400	287	1	107	5	6.68	< 0.001
CA	FD	200	162	22	10	6	5.99	< 0.01
TN	LD	400	378	3	19	0	0	*NS*
TN	FD	200	193	2	5	0	0	*NS*

*CA–California, USA; TN–Tennessee, USA; MO–Missouri, USA.

^A^Data presented in this table was quantified across collection locations and walnut branch section sample types whether or not samples were derived from lesion-directed or feature-directed methods.

^B^Number of individual samples.

^C^Pearson’s Chi-square test (α = 0.05) with Yates’ continuity correction.

^D^LD = lesion-directed drill sampling; FD = feature-directed drill sampling.

^E^*NS* = not statistically significant.

Pearson’s Chi-square test with Yates’ continuity correction was performed to determine if presence of *G*. *morbida* only, presence of *P*. *juglandis* only, or presence of both species was associated on a tree basis (Table 2). Drill sampling outcomes were pooled for each tree, and if any drill sample was positive, then this led to the decision that the tree was positive for TCD. Both drilling methods were effective at detecting one or more of the TCD organisms in the California and Tennessee locations [*X*^2^ (df = 1, n = 200) = 12.11, *P* < 0.001] (Table 2). Under the pooled data scenario and when the two drilling methods were analyzed separately, both lesion-directed [*X*^2^ (df = 1, n = 120) = 7.69, *P* = 0.005] and feature-directed [*X*^2^ (df = 1, n = 80) = 6.79, *P* = 0.009] drilling methods were efficient molecular means of detecting TCD (Table 2).

**Table 2:** Comparisons of molecular detection of *Geosmithia morbida* (*Gm*) and *Pityophthorus juglandis* (*Pj*) in any of the drilled samples (per tree) confirmed that a sample (tree) is positive for either organism^A^.

Location	Sample Type	N^B^	*Pj*^(-)^/*Gm*^(-)^	*Pj* ^((+)^/*Gm*^(-)^	*Pj* ^((-)^/*Gm*^(+)^	*Pj* ^((+)^/*Gm*^(+)^	*Χ*^2^ (df = 1)^C^	*P-*value
Combined thousand cankers disease-infested localities^D^								
CA, TN*	LD & FD ^E^	200	135	7	45	13	12.11	< 0.001
CA, TN	LD	120	76	1	36	7	7.68	0.005
CA, TN	FD	80	59	6	9	6	6.79	0.009

*CA–California, USA; TN–Tennessee, USA.

^A^Data presented in this table was quantified across collection locations and walnut branch section sample types whether or not samples were derived from lesion-directed or feature-directed methods.

^B^Number of individual samples.

^C^Pearson’s chi-square test (α = 0.05) with Yates’ continuity correction.

^D^Tallied observations across 10 lesion-directed or 5 feature-directed drill cores per tree.

^E^LD = lesion-directed drill sampling; FD = feature-directed drill sampling.

The logistic mixed model revealed that the California samples were seven times more likely to result in positive confirmation of TCD organisms when compared to Tennessee samples (contrast estimate 1.94, *P* < 0.001). The model also indicated that lesion-directed drilling was almost 13 times more likely to yield a positive confirmation of *G*. *morbida* from California-sourced *J*. *hindsii* samples than from Tennessee *J*. *nigra* samples (contrast estimate 2.55, *P*<0.001). In addition, among California *J*. *hindsii* trees sampled, *G*. *morbida* detection was about seven times more likely when using lesion-directed sampling than feature-directed sampling (contrast estimate 1.92, *P* < 0.001).

For *G*. *morbida*, we did not detect any cross amplification of DNA for GS004 across 100 different fungal isolates known to co-inhabit *P*. *juglandis* galleries (Table A in S1 File). In addition, our preliminary data indicated lack of cross-amplification for the following *Geosmithia* spp. (*G*. *argillacea*, *G*. *cylindrospora*, *G*. *lavendula G*. *namyslowski*, *G*. *pallida* sensu stricto, *G*. *putterillii*, *G*. sp. 2, *G*. sp. 10, *G*. sp. 21, *G*. sp. 23, and *G*. sp. 41) using previously developed microsatellite loci [31]. DNA from twenty-four bark or woodboring beetle species, three *Pityophthorus* spp. (*P*. *lautus*, *P*. *liquidambarus*, and *P*. *pulicarius*), and two *Monarthrum* spp. ambrosia beetles (*M*. *fasciatum* and *M*. *mali*) was amplified across *P*. *juglandis* microsatellite loci (Table B in S1 File). No other DNA samples from individuals that we tested were amplified across any of the 18 microsatellite loci used for analysis of cross-transferability [32]. Further, none of these samples amplified across WTB 192, which was the microsatellite locus used in the current study. DNA from the two *Monarthrum* spp. amplified across only one microsatellite locus (WTB12) (Table B in S1 File); DNA from *P*. *lautus* amplified across three loci (WTB 2, 13, and 19); DNA from *P*. *liquidambarus* amplified across six loci (WTB 1, 12, 13, 19, 128 and 130); and DNA from *P*. *pulicarius* amplified across eight loci (WTB 1, 2, 12, 13, 14, 19, 128 and 130).

## Discussion

Here, we report an efficient, sensitive, and cost-effective tool for the rapid molecular detection of TCD organisms. The detection of *P*. *juglandis* by this method may have been limited by various factors (see below), and identification of *P*. *juglandis* is a relatively quick procedure once a specimen is delivered to an expert, so much of the efficacy of our method is a consequence of improvements in the detection of *G*. *morbida*. The method significantly reduces the time required to detect *G*. *morbida* from weeks to only 4–6 hours and facilitates the process by using minute amounts of DNA taken from mixed samples of phloem tissue potentially containing host plant, pathogen(s), and/or vector(s). Besides reducing the time involved in the traditional culture-based approaches, the protocol developed here overcomes additional challenges when isolating and identifying *G*. *morbida*. These include the lack of a *G*. *morbida*-specific nutrient medium and the slow fungal growth rate, the latter resulting in competition in culture from frequently co-inhabiting and faster-growing fungi such as *Aspergillus* spp., *Fusarium* spp., *Penicillium* spp., and *Trichoderma* spp. Furthermore, potential *G*. *morbida* isolates generally must be subjected to several subculturing cycles into fresh nutrient media-plates before an axenic colony is achieved. This is especially true for samples obtained from the eastern USA [33] and from samples that are lightly infested by the vector.

The rapid molecular detection protocol described here revealed that branch sections from 34 out of 40 trees sampled from California were positive for the presence of *G*. *morbida* and/or *P*. *juglandis*, when data were pooled from lesion-directed and feature-directed sampling approaches. However, only 17 out of 40 branch section samples from Tennessee trees were assessed as positive. These findings likely reflect the difference in disease incidence that has been reported between regions [15]. The low detection of TCD in branch sections from Tennessee is not necessarily due to the inability of the protocol to provide detection of *G*. *morbida* and/or *P*. *juglandis*. It is, however, a reflection of the current incidence of the disease in Tennessee and provides evidence that variation in disease distribution is likely to occur within a site and even throughout portions of a tree. Samples from Tennessee were collected from trees growing in seven different counties in which TCD has been reported. However, not all trees within these areas exhibited symptoms and signs of TCD (i.e., likely not a 100% infection rate). On the other hand, California samples were taken from a single location in Yolo County, where the incidence and severity of TCD are high.

To address issues of false positive results, which could impact the validity and sensitivity of the detection method, we used the following approaches: (1) we tested the primers for the *G*. *morbida*-specific microsatellite locus GS 004 against several fungi that are found frequently within *P*. *juglandis* galleries, and belong to genera found frequently as endophytes or secondary pathogens of hardwood trees; and (2) we tested the *P*. *juglandis*-specific primer set against a group of insects that frequently colonizes *J*. *nigra*, including three different *Pityophthorus* species. In our preliminary studies, the *G*. *morbida*-specific microsatellite locus GS 004 has demonstrated lack of cross-transferability to other closely related *Geosmithia* species, thus mitigating the likelihood of false positive results.

Although there are over 220 *Pityophthorus* species described from North and Central America [40], here we tested only *P*. *lautus* Eichhoff, *P*. *pulicarius* Zimmermann, and *P*. *liquidambarus* Blackman, from which viable DNA could be extracted, across all *P*. *juglandis*-specific microsatellites [32]. *Pityophthorus lautus* has been reported from *J*. *nigra* and is frequently confused with *P*. *juglandis* in detection traps [24, 41], making detection more challenging. These species did amplify across 8 other microsatellite loci tested: *P*. *lautus* to WTB 2, 13 and 19; *P*. *pulicarius* to WTB 1, 2, 12, 13, 14, 19, 128, and 130; and *P*. *liquidambarus* to WTB 1, 12, 13, 19, 128, and 130 (Table B in S1 File). However, our diagnostic locus WTB 192 was not amplified in any of these species.

Branch sections from Missouri (hypothesized as negative controls) produced a positive amplification of WTB 192 in a few instances. To follow-up on these results, the PCR reaction was repeated, and during the summer of 2016 pheromone-baited Lindgren funnel traps were deployed in the Missouri orchards where the samples had been collected. After 3 bi-monthly collections, no *P*. *juglandis* were trapped. This supports the practical utility and specificity of this microsatellite locus for *P*. *juglandis*, but does not explain why several branch section samples were positive for *P*. *juglandis* in Missouri when no beetles were recovered from this site and there is no evidence for TCD in the area. Still, the putatively false positive result from Missouri could be explained by cross-transferability of WTB 192 into another closely related and frequently trapped *Pityophthorus* species that, due to lack of available specimens, we have been unable to test in our study (e.g., *P*. *crinalis* Blackman, Seybold et al. 2013b).

The ultimate goal of an early detection method is to be able to determine the presence of a pathogen or vector from asymptomatic plant hosts [42]. To simulate this, we tested feature-directed drilling methods that were conducted without requiring bark removal or closer examination of the host to detect cankers. Our results indicated that 37.5% of *J*. *hindsii* trees in California and 15% of *J*. *nigra* trees in Tennessee contained *G*. *morbida* and/or *P*. *juglandis*. Although the success of the feature-directed drilling method is not as robust as the lesion-directed drilling method, it might still be used to detect the presence of TCD organisms, particularly if aggregated sampling is employed (combining outputs from multiple drill samplings from a tree into a single DNA extraction product).

It appears that it was more difficult to detect the presence of *P*. *juglandis* (than *G*. *morbida*) when using the molecular methods with our samples. A likely explanation for this limited detection rate could be the low quality and quantity of *P*. *juglandis* DNA in the test samples. The quantity in the samples may have been below the detection threshold of the *P*. *juglandis*-specific primer. DNA quantities are expected to be low in the wood shaving samples because the DNA would have come from molted exoskeletal structures or frass from the beetles [26]. Another possible explanation for the low detection rate of *P*. *juglandis* is that other vectors may have been involved in our samples. *Geosmithia morbida* has been associated almost exclusively with *P*. *juglandis* [2], but the weevil *Stenomimus pallidus* Boheman and two ambrosia beetle species, *Xylosandrus crassiusculus* Motschulsky and *Xyleborinus saxesenii* (Ratzeburg), are also potential vectors of the pathogen [43, 44]. The interaction of plant host with our method may also have influenced our TCD detection results, with *J*. *hindsii* sampled in California vs. *J*. *nigra* sampled in Tennessee.

Utley et al. [8] tested the virulence of *G*. *morbida* on several *Juglans* species, including *J*. *hindsii* and *J*. *nigra*. Responses to *G*. *morbida* inoculations differed, indicating a higher susceptibility to canker development in *J*. *nigra* when compared to *J*. *hindsii* [8]. The authors also concluded that genetic variability among half-sib families of *J*. *nigra* influenced their level of resistance to *G*. *morbida*. Genetic variation among individuals of *J*. *hindsii* was not tested [8]. However, differences in TCD detection in California vs. Tennessee may have been impacted more by relative differences in population density of *P*. *juglandis* in the two areas, which have been considerably higher in California than in Tennessee for the past few years or by differences in the affinity of *P*. *juglandis* for *J*. *hindsii* vs. *J*. *nigra* in terms of host selection behavior [45].

Our rapid molecular detection method will facilitate the detection of TCD organisms from new suspect sites and will aid in monitoring disease persistence in existing quarantine and buffer areas, thus mitigating further disease spread. Although regulatory quarantines can be effective, this measure has not prevented TCD from spreading across the USA and to Europe [12, 20, 46]. Lack of quarantine success could have been a consequence of a limited capability for detecting and restricting infected materials at borders and control points. To reduce risk of TCD spread into Canada, national plant protection organizations have developed a real-time PCR-based detection method for *G*. *morbida* [27]. However, the method is based on traditional culturing protocols and utilizes Taq-Man technology focusing on a region of the β-tubulin gene as a DNA marker [27]. Their method lacks confirmation capability for the insect vector (*P*. *juglandis*) and does not enable detection following direct extraction of the fungus (*G*. *morbida*) from infested tree branches. Consequently, detection exclusively by their approach remains challenging and retains cumbersome steps including the need to obtain pure *G*. *morbida* cultures. The molecular method proposed here is relatively inexpensive, with an estimated per sample cost of $10.76 that could be further reduced if the method is optimized for the visualization of PCR amplicons through conventional gel electrophoresis.

In conclusion, the PCR-based protocol proposed in this study provides an efficient, sensitive, and cost-effective tool for the rapid molecular detection of the organisms that cause TCD. Confirmation time is reduced significantly from weeks to hours by using minute amounts of DNA obtained directly from infected material. Using species-specific microsatellite loci, we were able to confirm the presence of the pathogen and insect vector alone or in combination (important because of some state-specific quarantine rules). This method can be replicated easily in other labs and can be used to help comply with quarantine regulations and aid in the early detection of TCD in new areas. The use of species-specific microsatellites and the newly developed protocol here can be expanded to other species systems, which could aid in preventing the spread of introduced and/or invasive pathogens.

## Supporting information

S1 File Table A. Cross-amplification of *Geosmithia morbida* specific microsatellite locus GS 004 across fungal isolates known to co-inhabit walnut tree galleries. Table B. Cross amplification of *Pityophthorus juglandis* (WTB) specific microsatellite loci across twenty-four woodborning beetle species that are closely related to *P*. *juglandis* or are associated frequently with walnut trees. Fig A. An example of QIAxcel gel view and electropherogram of positive samples using GS 004 (upper) and WTB 192 (lower) microsatellite loci.
(XLSX)
